# Non‐islet‐cell tumor hypoglycemia as first manifestation of an advanced hepatocellular carcinoma

**DOI:** 10.1002/ccr3.5012

**Published:** 2021-10-23

**Authors:** Imen Rojbi, Wiem Ben Elhaj, Nadia Mchirgui, Meriem Jrad, Ibtissem Ben Nacef, Karima Khiari

**Affiliations:** ^1^ Endocrinology Charles Nicolle Hospital Tunis Tunisia; ^2^ Faculty of Medicine of Tunis University of Tunis El Manar Tunis Tunisia; ^3^ Medical Imaging Charles Nicolle Hospital Tunis Tunisia

**Keywords:** hepatocellular carcinoma, hypoglycemia, IGF‐2, NICTH

## Abstract

In a nondiabetic patient, fasting hypoglycemia is uncommon and warrants careful assessment. Although rare, NICTH should be considered in patients with recurrent hypoglycemia especially in those with risk factors for HCC.

## Graphical abstract

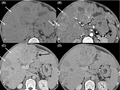

## 1 INTRODUCTION

Non‐islet‐cell tumor hypoglycemia (NICTH) is a rare but severe complication of malignancy. We present the case of 55‐year‐old man who was admitted for severe hypoglycemia. The diagnosis of insulinoma was ruled out. After clinical work‐ups, we made the diagnosis of metastatic HCC with production of IGF‐2. Tumor‐induced hypoglycemia (TIH) is rare and is almost due to excessive secretion of insulin by the tumor. However, TIH has been recently associated with the non‐islet‐cell tumor hypoglycemia (NICTH) syndrome.^1^ The underlying mechanism of hypoglycemia is production of incompletely processed insulin‐like growth factor 2 (IGF‐2).

Although the actual prevalence is not known, NICTH occurs in patients with solid tumors of mesenchymal or epithelial origin. For epithelial tumors, hepatocellular carcinoma (HCC) is the most frequent.^2^

In HCC, hypoglycemia is usually attributed to hepatocellular insufficiency at an advanced stage of the disease.

Rarely, NICTH has been observed as the initial manifestation of HCC.^3^

We present a patient who presented with severe hypoglycemia and was diagnosed with advanced HCC producing IGF2.

## 2 CASE PRESENTATION

A 55‐year‐old man presented to the Emergency Department with sudden loss of consciousness. His blood glucose level was 20 mg/dl. He was immediately resuscitated with intravenous dextrose infusion, and his mental status improved to baseline with adequate increase in blood glucose. The patient reported dizziness, weakness, and blurred vision preceding his loss of consciousness. This symptomatology occurred for 1 month twice a week.

Past medical history included only schizophrenia treated with Haloperidol and Trihexyphenidyl since 25 years. The patient had history of chronic alcohol use but he did not drink alcohol since 1 month. He was lethargic, and his BMI was 18.2 kg/m^2^. Clinical examination noted a hard and an irregular hepatomegaly with collateral venous circulation. No splenomegaly was found. His cardiovascular and respiratory system findings were within normal limits.

Laboratory parameters are shown in Table 1. During the hypoglycemic episode, we measured insulin, C‐peptide, and cortisol. Drug screens for sulfonylureas and other insulin secretagogues were negative.

**TABLE 1:** Laboratory data of the reported patient

Laboratory parameters	Unit	Measured value	Normal range
Serum glucose	mg/dl	20	70–100
Serum insulin	mUI/L	<0.1	3–10
Cortisol	nmol/L	526	101–535
Serum bilirubin	µmol/L	80	3.4–20
ALT	UI/L	71	6–55
AST	UI/L	243	5–34
ALP	UI/L	260	40 −150
Albumin	g/L	33	35–50
Serum cholesterol	mmol/L	5	3.2–5.18
Prothrombin time	s	16.5	9–11
Hemoglobin	g/dl	12	13–18
IGF−1	ng/ml	<4	71–263
IGF−2	ng/ml	561	396–1039
Serum AFP	UI/ml	>4000	<4

Abbreviations: AFP, alpha fetoprotein; ALP, alkaline phosphatase; ALT, alanine transaminase; AST, aspartate aminotransferase; IGF, insulin growth factor.

Abdominal ultrasound revealed enlarged dysmorphic liver, seat of multiple nodules saving no segment.

**FIGURE 1:**
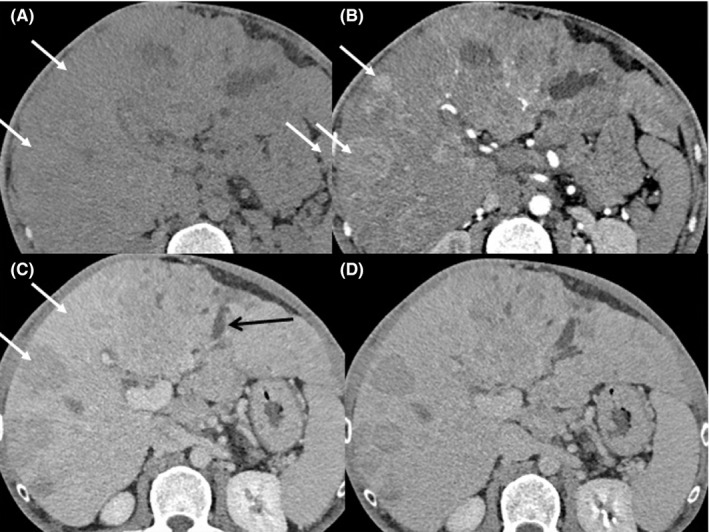
Multiphase abdominal computed tomography: The liver parenchyma is dysmorphic with irregular surface, portal vein enlargement and portosystemic collaterals. Numerous hepatic nodules and masses (White Arrow) isodense before contrast (A), enhancing vividly during arterial phase (B) and then washes out rapidly, becoming indistinct or hypoattenuating in the portal venous phase (C) with enhancing capsule in the late venous phase (D). They are associated with perfusion abnormality (Arrow head) due to arterioportal shunts: Multiple hepatocellular carcinoma

Computed tomography (CT) revealed multiple hepatic lesions, with the largest measuring 50 × 45 mm in the segment VIII of the liver. The lesions showed heterogeneous arterial enhancement with portal/delayed phase washout, consistent with multifocal multicentric HCC. The portal vein was dilated and multiple pulmonary metastases were present (Figure 1).

Tests for hepatitis B and hepatitis C were negative. Serum α‐fetoprotein was more than 4000 UI/ml (normal range, <4 UI/ml).

The diagnosis of HCC due to alcoholic cirrhosis was formed.

During his hospital stay, the patient presented many episodes of hypoglycemia (33– 64 mg/dl) requiring continuous intravenous dextrose infusion.

The patient's insulin level was <0.1 mIU/L, and the blood cortisol level was elevated, thus excluding, respectively, the diagnosis of insulinoma and adrenal insufficiency.

Further investigations showed insulin‐like growth factor 1 (IGF1): <4 ng/ml (normal 71–263), and IGF2: 561 ng/ml (normal 396–1039). The ratio IGF2/IGF1 was very high (>140).

Therefore, the diagnosis of HCC and NICTH with production of IGF‐2 was considered. Initially, the patient was treated with continuous 10% dextrose infusion. Due to the advanced disease stage and the general condition of the patient, he was not a candidate for surgical or other tumor‐directed therapies.

He received only symptomatic treatment, including oral prednisolone 30 mg once daily in addition to frequent high complex carbohydrate meals with some improvement in the severity of hypoglycemia. Then, he opted to pursue home hospice and passed away.

## 3 DISCUSSION

Non‐islet‐cell tumor hypoglycemia is the syndrome of hypoglycemia associated with any tumor other than an insulinoma. This syndrome is relatively uncommon and may be under‐diagnosed.

It is explained by overproduction by the tumor of incompletely processed forms of IGF‐2: pro‐IGF‐2. Both IGF‐2 and pro‐IGF‐2 stimulate insulin receptors, resulting in increased glucose utilization. Pro‐IGF‐2 and IGF‐2 suppress not only insulin but also growth hormone, which likely exacerbate hypoglycemia.^1^, ^2^, ^4^

In a non‐diabetic patient, fasting hypoglycemia is rare and warrants thorough evaluation.

This case is about a psychiatric patient with long history of alcohol use who presented with severe hypoglycemia.

Drug‐induced hypoglycemia was first considered. However, serum insulin was suppressed, thus excluding self‐administration of insulin secretagogues.

Ethanol inhibits gluconeogenesis in the liver, contributing to hypoglycemia but our patient did not drink alcohol since 1 month. In addition, the frequency of hypoglycemia did not improve during hospitalization. Therefore, alcohol or drug‐induced hypoglycemia was considered unlikely.

Furthermore, during hypoglycemia, cortisol level was normal and insulin level was very low excluding, respectively, the diagnosis of adrenal insufficiency and insulinoma.

In addition to the presence of a metastatic liver tumor, IGF‐I was suppressed, and IGF‐2 to IGF‐1 ratio was elevated. Given these findings, the diagnosis of NICTH due to HCC was made.

The diagnosis of NICTH is suspected when hypoglycemia is associated with low insulin and C‑peptide levels together with an inappropriate increase in the ratio of IGF‑2 to IGF‑1. Total IGF‐2 may be elevated or normal, but levels of pro‐IGF 2 are elevated. Measuring pro‐IGF‐2 in clinical practice is difficult. Unfortunately, it is not available in our country.

The ratio of IGF‐2/IGF‐1 is helpful when levels of IGF‐2 are normal like for our patient. A ratio greater than 10: 1 is virtually diagnostic.^2^, ^5^, ^6^, ^7^

In about half of patients with NICTH, hypoglycemia present, often long, after the diagnosis of the neoplasm. About half of them present with hypoglycemia as first manifestation of the disease.^2^

Unfortunately, our patient had presented late at an advanced stage of his disease with severe hypoglycemia.

The present patient had hepatocellular carcinoma with a liver failure. We suggested that in addition to NICTH, the decrease in glycogen storage due to the decreased liver function had made hypoglycemia more frequent and severe.

In fact, hypoglycemia in HCC is explained by two mechanisms. The most common is hepatocellular insufficiency seen at the late stage of the disease. More rarely and from the early stage, hypoglycemia may be related to a paraneolplastic syndrome linked to the production of IGF2 or Pro‐IGF2.^3^

Initial treatment of NICTH aims at maintaining euglycemia. Most of these patients are managed with parenteral dextrose infusion. However, the majority of them fail to maintain adequate glucose levels and require a second modality to treat hypoglycemia with varying degrees of success.

The most effective treatment of hypoglycemia with IGF‐2 producing tumor remains surgical resection.^2^, ^8^ Chemotherapy, radiation, and selective embolization are other therapeutic options reported for patients with advanced disease.^9^

When hypoglycemia persists after surgery, radiation, and/or chemotherapy, multiple palliative treatments have been employed. Glucocorticoids have been the most effective agents. They prevent hypoglycemia by increasing gluconeogenesis, inhibition of peripheral glucose uptake, and modulating the growth hormone‐IGF axis. Glucocorticoids also have been shown to decrease the production of pro‐IGF‐2. But, the effects of glucocorticoids vary among patients, requiring close titration, and they are ineffective after reduction or cessation of therapy.^2^, ^10^

Our patient was put on prednisolone 30 mg per day and 2–3 hourly frequent high‐carbohydrate feeds.

Other possible treatment options include Recombinant GH, somatostatin analogs, diazoxide and glucagon.^2^, ^4^

In conclusion, there is a well‐established association between solid tumors and production of IGF‐2. Although rare, NICTH should be considered in patients with recurrent hypoglycemia especially in those with risk factors for HCC.

## CONFLICT OF INTEREST

There is no conflict of interest.

## AUTHOR CONTRIBUTIONS

Wiem Ben Elhaj: involved in conception and design of study, literature search, and drafting of article. Imen Rojbi, and Ibtissem Ben Nacef: involved in design of manuscript, literature search, and drafting of article. Myriam Jrad: involved in radiographic image acquisition and interpretation. Karima Khiari: involved in design of manuscript, drafting of article, and final approval. All authors read and approved the final version of the manuscript.

## ETHICAL APPROVAL

Written informed consent was obtained from the patient.

## CONSENT

The authors have confirmed during submission that patient consent has been signed and collected in accordance with the journal's patient consent policy.

## Data Availability

The data of this case are available from the corresponding author upon reasonable request.
